# Vulnerability of Protoxylem and Metaxylem Vessels to Embolisms and Radial Refilling in a Vascular Bundle of Maize Leaves

**DOI:** 10.3389/fpls.2016.00941

**Published:** 2016-06-27

**Authors:** Bae Geun Hwang, Jeongeun Ryu, Sang Joon Lee

**Affiliations:** Center for Biofluid and Biomimic Research, Department of Mechanical Engineering, Pohang University of Science and TechnologyPohang, South Korea

**Keywords:** dehydration, embolism, radial refilling, X-ray imaging, xylem transport

## Abstract

Regulation of water flow in an interconnected xylem vessel network enables plants to survive despite challenging environment changes that can cause xylem embolism. In this study, vulnerability to embolisms of xylem vessels and their water-refilling patterns in vascular bundles of maize leaves were experimentally investigated by employing synchrotron X-ray micro-imaging technique. A vascular bundle in maize consisted of a protoxylem vessel with helical thickenings between two metaxylem vessels with single perforation plates and nonuniformly distributed pits. When embolism was artificially induced in excised maize leaves by exposing them to air, protoxylem vessels became less vulnerable to dehydration compared to metaxylem vessels. After supplying water into the embolized vascular bundles, when water-refilling process stopped at the perforation plates in metaxylem vessels, discontinuous radial water influx occurred surprisingly in the adjacent protoxylem vessels. Alternating water refilling pattern in protoxylem and metaxylem vessels exhibited probable correlation between the incidence location and time of water refilling and the structural properties of xylem vessels. These results imply that the maintenance of water transport and modulation of water refilling are affected by hydrodynamic roles of perforation plates and radial connectivity in a xylem vascular bundle network.

## Introduction

Plants transport water and dissolved minerals through xylem vessels composed of dead lignified cells (Zimmermann and Brown, 1971; Holbrook et al., 2002). Plants are easily vulnerable to environmental changes because of their inherent passivity and immobility. However, they have adapted to harsh environments and survived with their own indigenous strategies, such as stomatal gating (Hetherington and Woodward, 2003; Raven, 2014), ion-mediated flow regulation through pit membranes (Zwieniecki et al., 2001b; Nardini et al., 2011), and axial and radial transport of water in redundantly interconnected xylem vessels (Tyree et al., 1994; Loepfe et al., 2007; Fan et al., 2009).

The structural characteristics of a xylem vessel network enables stable water transport in plants although individual xylem vessels are vulnerable to embolism. Xylem networks have been evolved to efficiently supply water to leaves and simultaneously protect against cavitation and spread of embolism (Sperry, 2003). Xylem vessels are also interconnected to provide redundancy in the network of hydraulic conduits, offering alternative pathways to bypass the embolized vessel element, and maintain stable water transport (Tyree et al., 1994; Loepfe et al., 2007).

Many studies have focused on the structural features of porous structures in xylem vessels of vascular plants and xylem vessel network in the last several decades. Flow resistance in the perforation plates of xylem vessels was experimentally measured and numerically simulated (Schulte et al., 1989; Schulte and Castle, 1993; Ellerby and Ennos, 1998; Schulte, 1999). Bordered intervessel pit membranes were also investigated in terms of air-seeding prevention (Melcher et al., 2003; Choat et al., 2004, 2005, 2008; Hacke et al., 2004; Sperry and Hacke, 2004; Meyra et al., 2007; Plavcová et al., 2013) and hydraulic resistance (Pittermann et al., 2005; Choat et al., 2008; Schulte, 2012; Schulte et al., 2015). Although the hydraulic permeability and radial transport in xylem vessel networks were experimentally investigated in a few studies (Zwieniecki et al., 2001a, 2003; Choat et al., 2006; Brodersen et al., 2013), most of the previous studies focused on the structural traits of porous vascular bundles (Konrad and Roth-Nebelsick, 2005; Choat et al., 2008; Schmitz et al., 2008; Jansen et al., 2009). Recently, the refilling kinetics in plants have been unveiled, by using non-destructive imaging techniques such as magnetic resonance imaging (Holbrook et al., 2001; Kaufmann et al., 2009; Zwieniecki et al., 2013; Fukuda et al., 2015) and X-ray imaging (Brodersen et al., 2010; Kim and Lee, 2010; Lee et al., 2013). However, relatively little is known about the dynamic process of water-refilling phenomena in relation to xylem structures.

In this study, synchrotron X-ray micro-imaging technique was employed to directly observe structural characteristics of xylem vessels in vascular bundles of excised maize leaves in relation to vulnerability to embolisms and water refilling with high spatial resolution. The reliability and accuracy of the current experimental methods (Melcher et al., 2012; Cochard et al., 2013; Sperry, 2013; Wheeler et al., 2013; Delzon and Cochard, 2014; Torres-Ruiz et al., 2015) using excised plant samples have been in debate; the cutting of xylem vessels entrains air into xylem vessels of vascular plants, disrupting water status in the excised samples. However, this study intentionally exposed the xylem vessels to air, for draining water-filled xylem vessels, and utilized the artificially embolized vascular structure of excised leaves as a platform to observe water-refilling process (Rolland et al., 2015). Thus, it did not need to concern about the artifacts caused by the induction of embolism in cutting plant samples. The consecutively recorded X-ray images of water-refilling dynamics directly showed temporal movements of discontinuous water columns in air-filled xylem vessels in a vascular bundle. The observed flow phenomena support the contribution of intervessel pathways to water-flow regulation and embolism repair in vascular plants. In addition, the hydrodynamic roles of perforation plates and radial connectivity of xylem vessels were considered based on the observed water-refilling phenomena during the rehydration process.

## Materials and methods

### Plant material

Wild-type maize (*Zea mays* L.) was used as a test plant in this experiment. Plant samples were hydroponically grown for 4–8 weeks in an environment-controlled facility at 25°C and 70% relative humidity, with 10 h of daily illumination (300 μmol PAR m^−2^ s^−1^). Photosynthetically active radiation (PAR) was monitored using a PAR irradiation sensor (E90, Jauntering International Corp., Taiwan). The plant samples were not exposed to any stress conditions before the leaf excision. The water potential would be ranged from −0.2 to approximately −1.0 MPa, in consideration of the corresponding value for leaves of well-watered herbaceous plants (Taiz and Zeiger, 2010). The stationary hydrostatic pressure in the parenchyma cells of *Z. mays* leaves is usually in the range of 0.2–1.0 MPa (Kim and Steudle, 2007) and its upper limit in well-watered plants is ~3 MPa (Taiz and Zeiger, 2010).

### Anatomical characterization of xylem using scanning electron microscopy (SEM)

Blades and sheathes of maize were cut into 1–2-mm-wide slices and fixed in 3% (v/v) glutaraldehyde, and then dehydrated using a series of ethanol gradients. After the ethanol solution was replaced with isoamyl acetate, the specimens were dried in a CO_2_ critical-point drying system (HCP-2, Hitachi, Japan). The dried samples were then coated with a thin layer of gold using an ion coater (PS-1200, PARAONE, South Korea). Sample images were captured by field-emission SEM (XL 30S FEG FE-SEM, Phillips, the Netherlands).

### Generation of embolisms and rehydration in excised leaves

Maize leaves were excised 10 cm from the tip of the leaf blades under water using a sharp razor blade. The excised leaf sample was vertically placed in a U-shaped sample holder sealed with Kapton tape. Xylem vessels in the excised leaf were dehydrated by being exposed to air at room temperature for 5 min afterward. After the dehydration process, the cut end of the leaf was immersed in water for 5 min by controlling the amount of water containing in the holder. The dehydration–rehydration procedure was repeated 5–6 times, until the water-refilling capability of the excised leaf sample deteriorated. The dehydration and rehydration phases were visually monitored at a position ~2–3 mm above the excised section of the sample using a 2D synchrotron X-ray micro-imaging technique.

### 2D synchrotron X-ray micro-imaging

Synchrotron X-ray imaging techniques were used to obtain phase-contrast images of the water-refilling behavior in the vascular bundles of maize leaves (Lee and Kim, 2008; Brodersen et al., 2010; Kim and Lee, 2010; Lee et al., 2013; Hwang et al., 2014; Ryu et al., 2014). This experiment was conducted using the 6D X-ray micro-imaging beamline of the Pohang Accelerator Laboratory. Temperature was maintained at 25°C under relative humidity of 39–57% and PAR intensity was maintained at 35 μmol PAR m^−2^ s^−1^ in the experimental hutch. Consecutive X-ray images were captured at a frame rate of 5 frames per second (fps) and an exposure time of 100 ms with a charged coupled device camera (Vieworks VH-11MC, Vieworks, South Korea) using a 10 × objective lens. A mechanical shutter and attenuation plates were utilized to minimize biological damage of the test samples caused by direct exposure of X-ray beam. The field of view (FOV) was 3600 × 2400 μm^2^, and the spatial resolution was 0.9 μm/pixel.

### Evaluation of xylem properties and refilling process in xylem vessels in 2D X-ray images

Xylem properties and the water contents of xylem vessels were measured using a digital image processing software (ImageJ, National Institutes of Health, USA; Schneider et al., 2012). The inner diameter of each xylem vessel was determined by averaging five measurements along the section of interest. The distinctive structures in the xylem vessels were designated as fixed reference points. This allowed for the estimation of relative height or displacement of water columns to evaluate the change in water columns refilled in empty xylem vessels. The volume of each water column was evaluated by reconstructing a simple 3D figure based on its 2D projected images. The 3D figure was a combination of spheres and bodies of revolution confined in a cylindrical vessel. Temporal variations of the refilling water columns were evaluated based on the frame rate of the consecutively recorded X-ray images. The volume flow rate was evaluated from the volumetric changes of each water column for a certain time interval. A xylem vessel that filled with water at less than 20% of its length, as observed in the FOV just after the dehydration process, was classified as an embolized vessel.

## Results

### Anatomical characteristics of a vascular bundle of maize

**Figure 1:**
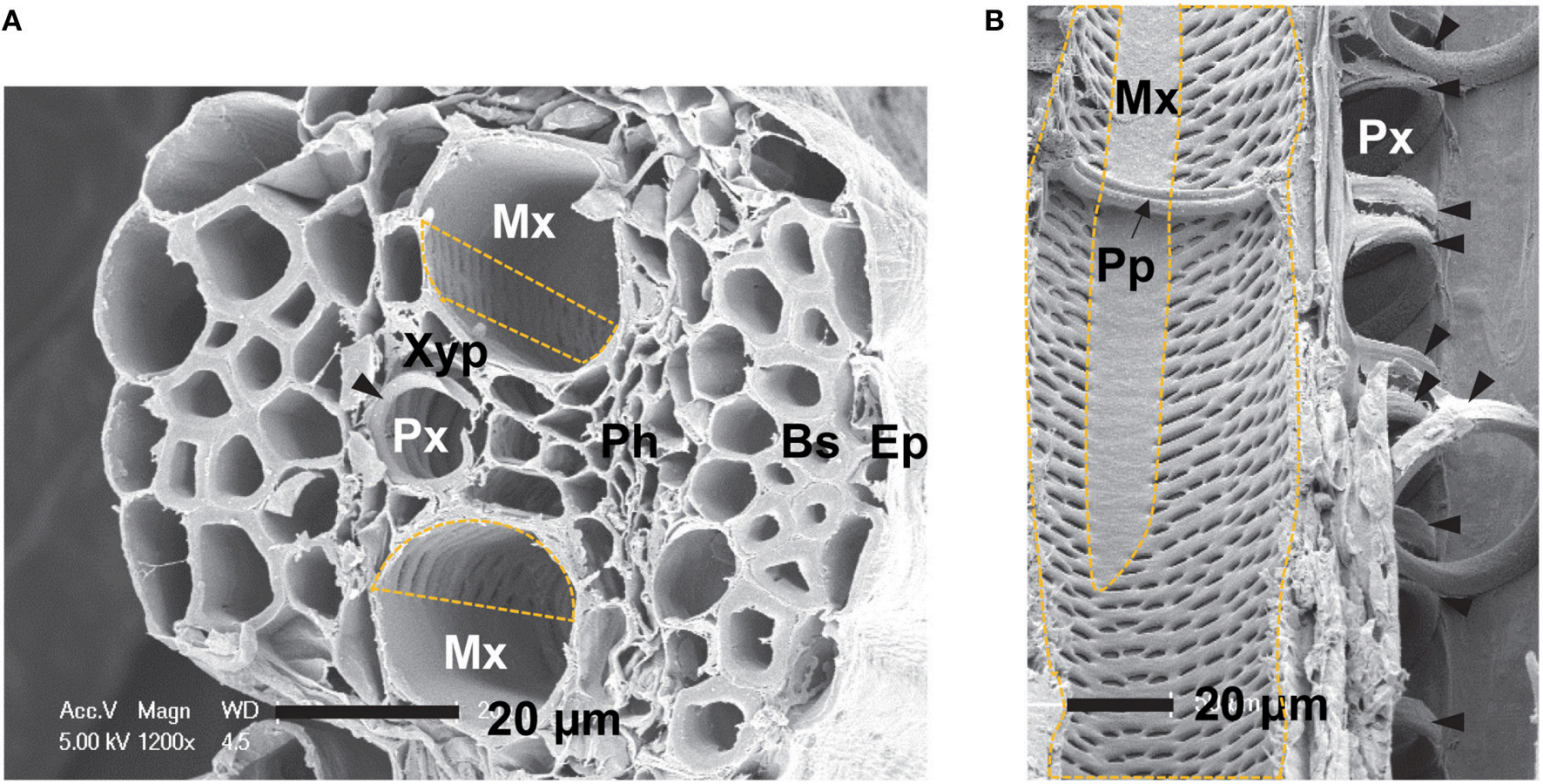
**SEM images of xylem vessels in a vascular bundle of maize (*Zea mays* L.). (A)** Transverse section of a maize sheath, showing a protoxylem (Px) vessel with secondary wall thickenings (filled arrowheads) and two metaxylem (Mx) vessels with pits distributed in a nonuniform manner (dashed box). **(B)** Longitudinal section of a maize leaf, showing protoxylem and metaxylem vessels with a single perforation plate (Pp). Ep, epidermis; Bs, bundle sheath fiber; Ph, phloem; Mx, metaxylem vessel; Px, protoxylem vessel; Xyp, xylem parenchyma cell; Pp, perforation plate. Scale bar = 20 μm.

A single vascular bundle consisted of a protoxylem (Px) vessel and two metaxylem (Mx) vessels in a maize sheath (Figure 1A). The protoxylem vessel had secondary wall with annular and helical thickenings. These thickenings were intermittently fused within a protoxylem vessel element (Figure 1B). The diameter of the protoxylem vessels was 10.9 μm. It was smaller than that of the metaxylem vessels of 23.6 μm (Figure 1A). The secondary cell walls of the metaxylem vessels had pits distributed nonuniformly, in particular, in the region near the protoxylem vessel (dashed lines, Figures 1A,B). The metaxylem vessel had simple perforation plates at certain intervals to regulate the axial flow (Figure 1B). The approximate distance between perforation plates in metaxylem vessels of maize leaves was 435 ± 127 μm (*n* = 10). Xylem parenchyma (Xyp) cells were distributed between the protoxylem and metaxylem vessels, and surrounded the xylem vessels (Figure 1A).

### Different embolization ratios according to xylem type

X-ray images of the protoxylem and metaxylem vessels in 10 vascular bundles of five maize plant leaves exhibited different vulnerability to embolisms caused by the dehydration process. Although the leaf cut ends of all maize leaf samples were exposed to air, not all of the protoxylem and metaxylem vessels were embolized during the dehydration–rehydration procedure. The embolization ratio in the protoxylem vessels with an average diameter of 18 μm, after applying the artificial dehydration process, was about 36.2% from 58 observations. In contrast, the embolization ratio was 86.2% from 174 observations in the metaxylem vessels with an average diameter of 26.7 μm.

### Discontinuous radial refilling in the embolized protoxylem and metaxylem vessels

Embolized protoxylem and metaxylem vessels in a vascular bundle were refilled with water, depending on the hydraulic status of the adjacent xylem vessels. Xylem vessels in a vascular bundle exhibited mutual interaction during water-refilling processes. Ten vascular bundles of dehydrated maize leaves were investigated in this study.

**Figure 2:**
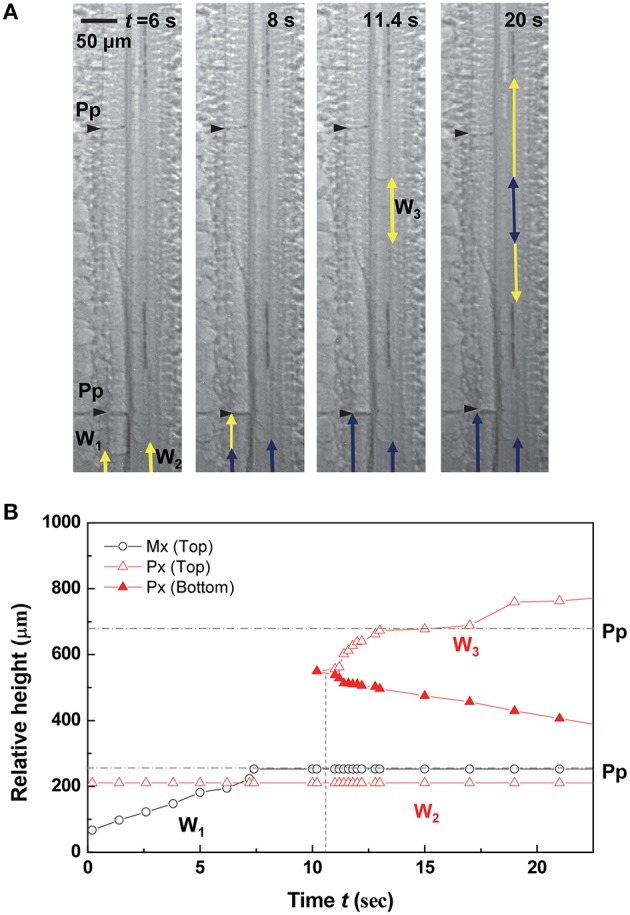
**Resumption of water refilling influenced by adjacent xylem vessel. (A)** Sequential kinetics of water refilling in an artificially embolized vascular bundle of a maize leaf in the 2nd dehydration/hydration cycle. **(B)** Temporal variations of the relative heights of three water columns W_1_, W_2_, and W_3_ from the bottom of field of view (FOV) image. Horizontal dotted lines indicated the height of perforation plates (Pp) on the left metaxylem vessel, and the vertical dotted lines indicated the times of W_3_ appearance, and W_1_ and W_2_ resumption.

**Figure 3:**
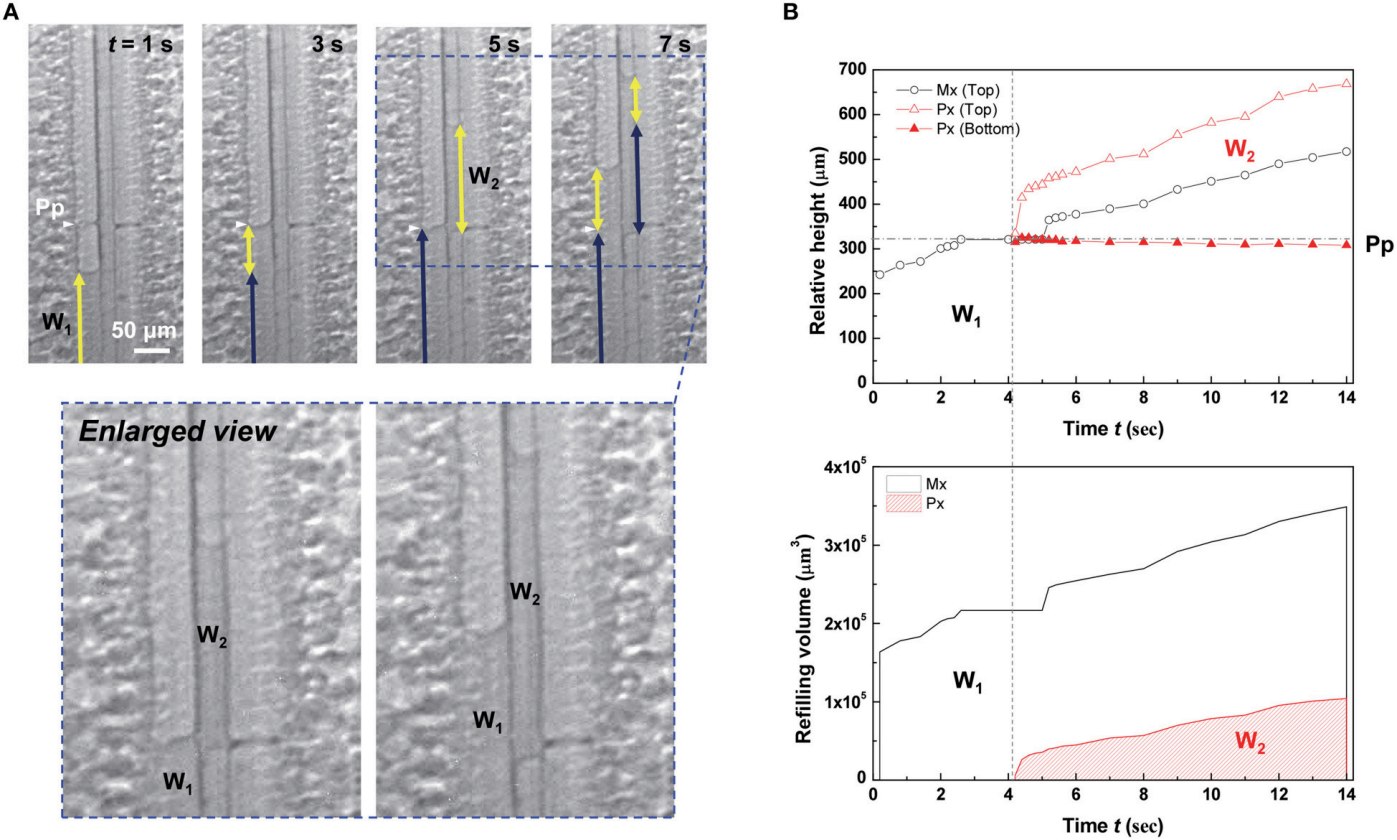
**Accompanied growth of water columns in refilling**. **(A)** Sequential kinetics of water refilling in an artificially embolized vascular bundle of a maize leaf sample in the 4th dehydration/hydration cycle. **(B)** Temporal variations of the relative height (upper) and volume (lower) of the two water columns W_1_ and W_2_. Horizontal dotted lines indicated the height of perforation plates (Pp) on the left metaxylem (Mx) vessel, and the vertical dotted lines represented the times appearing the discontinuous water column W_2_.

**Figure 4:**
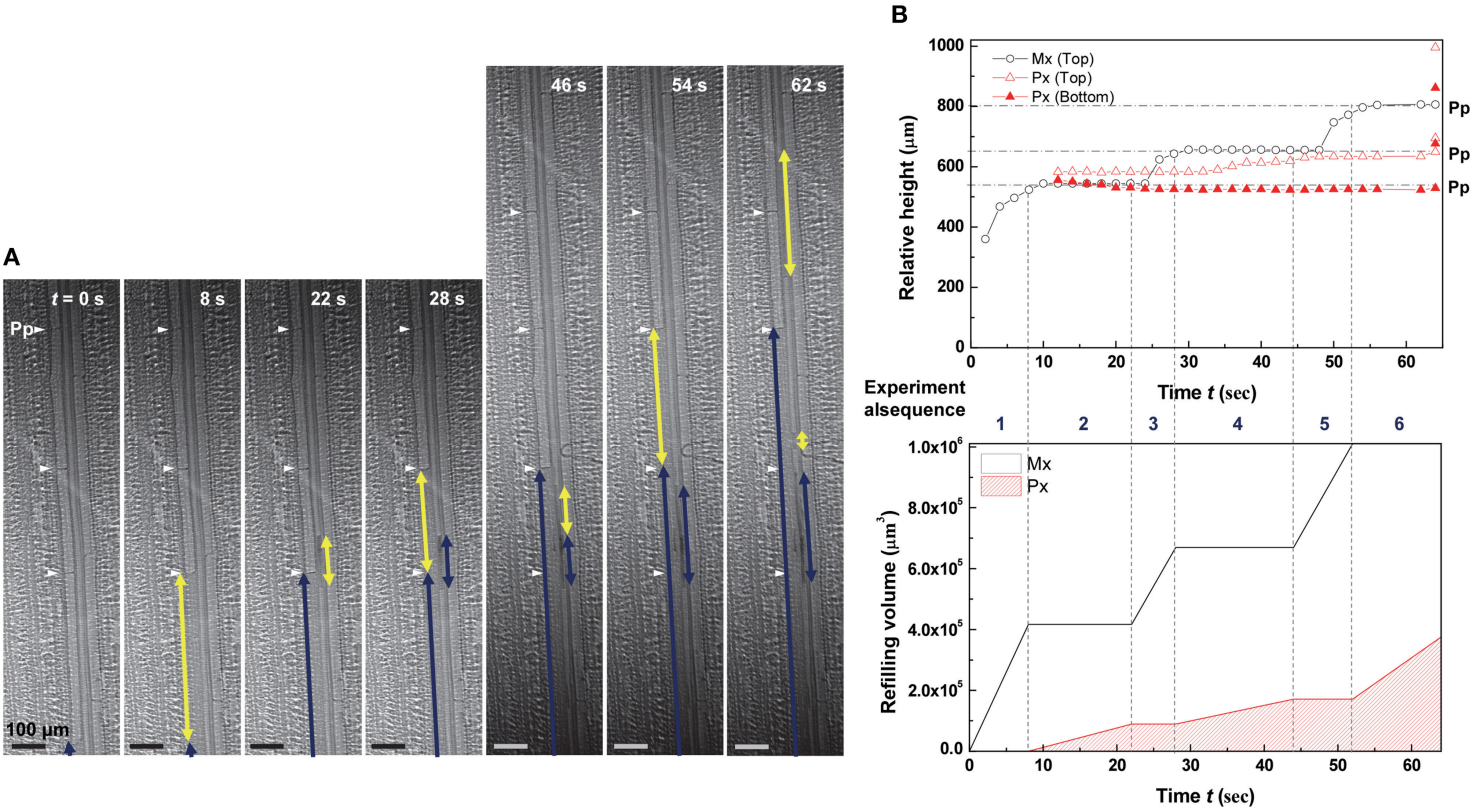
**Ping-pong pattern of water refilling in embolized metaxylem and protoxylem vessels of a vascular bundle**. **(A)** Sequential kinetics of water refilling in embolized metaxylem (Mx) and protoxylem (Px) vessels of an excised maize leaf in the 1st dehydration/hydration cycle. **(B)** Temporal variations of the relative heights (upper) and volume (lower) of refilling water in the water columns. Horizontal dotted lines indicated the height of perforation plates (Pp) on the left metaxylem vessel, and the vertical dotted lines represented the times of growth between the two xylem vessels by turns.

**Figure 5:**
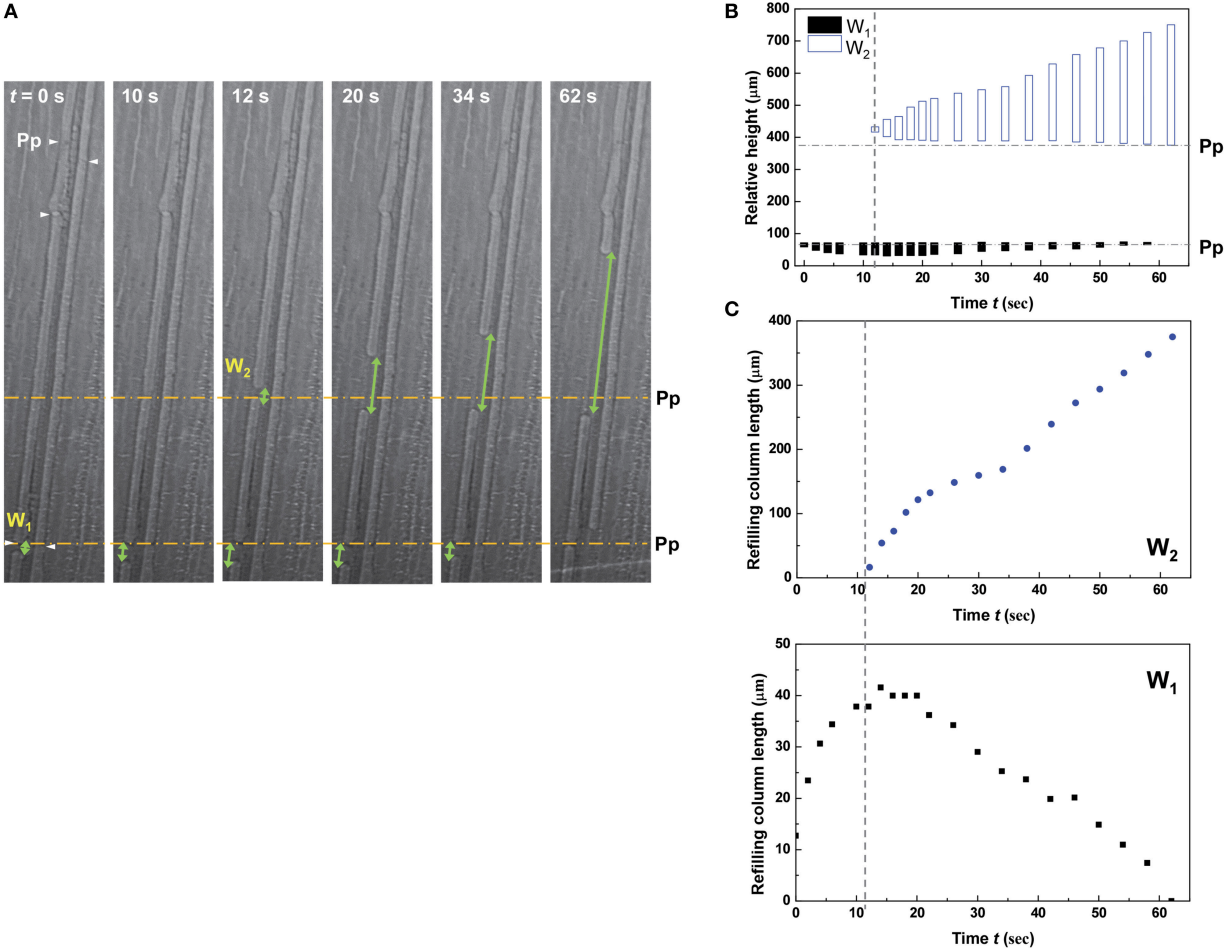
**Interdependent change of two discontinuous water columns in a metaxylem vessel during refilling. (A)** Sequential kinetics of water refilling in embolized metaxylem vessels and a water-refilled protoxylem vessel of an excised maize leaf in the 5th dehydration/hydration cycle. **(B)** Temporal variations of the relative heights of two discontinuous refilling water columns W_1_ and W_2_. **(C)** Temporal variations of the length of W_1_ and W_2_.

The stoppage of water-refilling phenomena occurred frequently at the perforation plates in metaxylem vessels, and the water refilling pattern was closely related to the water status of adjacent vessels in a vascular bundle (Figures 2–**5**). Figure 2 shows that the water column W_1_ in the left embolized metaxylem vessel ascended for the first 8 s, while the water column W_2_ did not move in the embolized protoxylem vessel. When the ascent of W_1_ stopped at the perforation plate, a discontinuous water column W_3_ burst out above W_2_ in the protoxylem vessel at *t* = 11.4 s. As the water column W_3_ expanded into the upper and lower sides, the growth of W_1_ and W_2_ stopped.

A discontinuous water column started to appear in the protoxylem vessel at the instant of water-refilling stoppage in the adjacent metaxylem vessel (Figure 3 and Movie S1). When the refilling water column W_1_ in the left metaxylem vessel reached a perforation plate, the ascending movement stopped at *t* = 3 s. When the ascent of W_1_ in the metaxylem vessel stopped for 1 s, the discontinuous water column W_2_ in the adjacent protoxylem vessel started to grow near the perforation plate. Thereafter, both xylem vessels were simultaneously refilled with water.

The embolized protoxylem and metaxylem vessels in a vascular bundle were refilled with water by turns (Figure 4 and Movie S2). The left embolized metaxylem vessel was refilled from the water reservoir below and it took 8 s for the refilling water column to reach a perforation plate. Water stopped rising in the metaxylem vessel (*t* = 8–22 s), but a short water column started to appear and grow at the embolized protoxylem vessel in the same vascular bundle. The growth of the water column in the protoxylem vessel stopped at *t* = 28 s, and the previously stopped water column in the metaxylem vessel started to rise up again toward the next upper perforation plate (*t* = 28–44 s). Water stoppage on one vessel was followed by resumption on the other previously stopped vessel. This ping-pong alternating pattern of water refilling in two adjacent xylem vessels was demonstrated well by their volume variations (Figure 4B). The characteristics of three representative water refilling phenomena observed in this study were summarized (Table 1).

**Table 1:** **Water refilling phenomena in the embolized protoxylem and metaxylem vessels of three representative vascular bundles in excised maize leaves**.

**Sample**	**Metaxylem (Mx) vessel**			**Protoxylem (Px) vessel**				**Mx**
	**Vessel diameter (μm)**	**Location of water stoppage (Relative height)**	**Water stoppage time (s)**	**Vessel diameter (μm)**	**Water refilling time (s)**	**Location of water influx**	**Mean speed (μm/s)**	**Mean refilling speed after water stoppage (μm/s)**
#1 (Figure 2)	35.5	Pp1: 257 μm	23	30.4	10.2–26 (during stoppage)	340 μm above Pp1	25.5 (upward & downward)	86.9 (at Pp1–Pp2)
#2 (Figure 3)	29.3	Pp1: 326 μm	2.4	19.2	4.2–5 (during stoppage)	Near Pp1	129 (upward)	
					5–14 (refilling in Px & Mx)	Near Pp1	26.4 (upward)	21.8 (at Pp1–Pp2)
#3 (Figure 4)	32.2	Pp1: 544 μm	14	26.6	10–24 (1st stoppage)	Near Pp1	2.2 (downward)	18.6 (at Pp1–Pp2)
		Pp2: 656 μm	18		28–46 (2nd stoppage)	Near Pp1	2.8 (upward)	18.7 (at Pp2–Pp3)
		Pp3: 806 μm	16		65–75 (3rd stoppage)	Near Pp2 and Pp4	1.7 (upward)	

### Refilling in embolized metaxylem and water-filled protoxylem vessels

Two discontinuous water columns W_1_ and W_2_ were subsequently formed and interdependently changed in a single embolized metaxylem vessel during water-refilling process, where the adjacent protoxylem vessel was previously fully refilled with water (Figure 5 and Movie S3). On the left metaxylem vessel, W_1_ was formed by radial refilling and began to grow. The radial refilling process started, near the perforation plate, 708 μm above the cut end of the test leaf. Approximately *t* = 12 s later, W_2_ appeared around another perforation plate located at a higher position in the same metaxylem vessel. It continued to grow, while W_1_ shrank instead and disappeared within 1 min. The water-refilling behaviors of W_1_ and W_2_ in the same metaxylem vessel showed a sort of temporal correlation.

## Discussion

Not all of the xylem vessels were embolized, and protoxylem vessels retained water with high probability, approximately twice more than metaxylem vessels, even though vascular tissues in maize leaves were excised and exposed to air. Protoxylem vessels mostly remain filled with water as non-conducting vessels. Vascular bundles composed of xylem vessels with various sizes and structures would be helpful for vascular plants to maintain hydraulic functions.

The interdependence of refilling of discontinuous water columns from radial water influx in both metaxylem and protoxylem vessels was also revealed in this study. Based on the water refilling patterns in the embolized vascular bundles, water moved from metaxylem to protoxylem vessels and vice versa (Figures 2–4). When two metaxylem and one protoxylem vessels in a vascular bundle were embolized, the water meniscus moved upward in one of the metaxylem vessels which was caused by capillary force. After the embolized metaxylem vessel was refilled with water, discontinuous water columns appeared and water-refilling phenomena started in the adjacent embolized protoxylem vessels. The water-refilled metaxylem vessel might play a role as a water-supplier to adjacent embolized vessels. The incidence of these discontinuous water columns in the embolized xylem vessels suggests that the radial water transport triggers water refilling for embolism repair. The radial water refilling was caused by osmotic flow from surrounding living cells (Holbrook and Zwieniecki, 1999; Brodersen et al., 2010; Secchi and Zwieniecki, 2011). However, this temporal correlation of the vascular bundle structures suggests the possibility of hydraulic connection between adjacent xylem vessels in a vascular bundle.

When metaxylem vessels were embolized and a protoxylem vessel was filled with water, discontinuous water columns appeared in the adjacent metaxylem vessel of the same vascular bundle. Interdependence of two isolated water columns in a metaxylem vessel (Figure 5) also supports the hydraulic connectivity in a vascular bundle. In addition, synchronized volume change of two water columns supports the possibility of a common water source for the radial influx into the metaxylem vessels. The water-filled protoxylem vessel in the same vascular bundle seems to work as a water supplier to the adjacent embolized metaxylem vessels (Figure 1).

The hydrodynamic connectivity of xylem vessels could be considered for two different cases for water-filled and embolized vessels. A multivessel vascular bundle system contains three main xylem vessels: two metaxylem vessels having a relatively large diameter, and one protoxylem vessel of small diameter (Figure 1). When there is no embolism, water is transported as a continuous flow through large metaxylem vessels in an efficient manner. The axial pathways of metaxylem vessels have the least hydraulic resistance, which can be easily estimated from Poiseuille's law. On the contrary, when there are embolized xylem vessels in a vascular bundle, water may be transported in both axial and radial directions through the most efficient local pathway into embolized xylem vessels.

The interconnected xylem vessels constitute a well-designed and optimized multivessel network. The xylem vessels have perforation plates and intervessel pit membranes; these porous structures make locally different hydraulic resistances for two-phase flows such as air-seeded liquid flow. For example, perforation plates have been known to act as pressure controllers in the hydraulic networks used for embolism repair (Lee and Kim, 2008; Kim and Lee, 2010; Lee et al., 2013). Interestingly, our findings demonstrate that water stoppage at the level of perforation plates in metaxylem vessels seemingly enables to favor a bypass through protoxylem vessels (Figures 2–5); the results give implications for hydrodynamic role of perforation plates to supply water into embolized vessels. Intervessel pit membranes between adjacent xylem vessels also function as smart valves (Holbrook and Zwieniecki, 1999; Zwieniecki and Holbrook, 2000; Meyra et al., 2007). The hydraulic connectivity of xylem vessels may coordinate the transmission of hydrostatic pressure through the porous morphological structures: the perforation plates and the spatial distribution of intervessel pit membranes.

Meanwhile, based on the overall spatial distribution of pits on the xylem wall of maize leaves (Figure 1), permeable vessels have a uniform hydraulic resistance over a wide range of vertical distances. This may result in large-scale radial water flow. However, the water refilling patterns and the locations of water influx (Table 1) indicated that the hydraulic connection between xylem vessels have specific dedicated pathways. Based on these results, radial refilling occurred at specific heights, mostly near the locations of perforation plates. To visualize the water-flow regulation through intervessel pit elements, bio-imaging techniques, including synchrotron X-ray microscopy, should be further advanced to the level of resolving dynamic flow phenomena at nanometer scale.

In addition, to measure the variations of xylem tension in individual xylem vessels would be helpful for elucidating water refilling dynamics examined in this study. The measurement of water potential in plant leaves (Scholander et al., 1965) cannot reflect the rapid changes in water contents within tens of seconds in individual xylem vessel where water is refilled. But, a cell pressure probe (Wei et al., 1999; Knoblauch et al., 2014) can be employed to measure rapid change in xylem pressure when water refilling stops near perforation plates and water is refilled in a radial direction from adjacent cells and vessels. It requires a remote manipulation of the pressure probe in a delicate manner with submicron accuracy to simultaneously observe water-refilling phenomena by using X-ray microscope.

In summary, the porous and permeable structures of xylem vessel wall and radial connectivity of protoxylem and metaxylem vessels in a vascular bundle play important roles in smart regulation of water transport and maintenance of hydraulic conductivity in vascular plants. The composition of xylem vessels having different diameters in a vascular bundle is helpful for reducing the danger of dehydration. In addition, the perforation plates and intervessel pits in xylem vessels enable discontinuous radial water refilling in the adjacent embolized xylem vessels and make hydraulic conductivity maintain. Detailed understanding on the hydrodynamic connectivity of xylem vessel network would provide a plausible mechanism for embolism repair in vascular plants, especially for fast and effective water refilling.

## Author contributions

BH and JR are equally contributed co-first authors. BH and JR prepared the sample, designed the experiments, performed the measurements, and analyzed the data. All authors discussed the results, wrote the paper, and commented on the manuscript.

### Conflict of interest statement

The authors declare that the research was conducted in the absence of any commercial or financial relationships that could be construed as a potential conflict of interest.

## Supplementary material

Movie S1 **Refilling process shown in Figure 3 (Total *t* = 14 s; × 1.0)**.

Movie S2 **Refilling process shown in Figure 4 (Total *t* = 76 s; × 2.0)**.

Movie S3 **Refilling process shown in Figure 5 (Total *t* = 62 s; × 4.0)**.
